# Supplementary material for: NanoMethViz: An R/Bioconductor package for visualizing long-read methylation data
Source: PLoS Comput Biol. 2021 Oct 25;17(10):e1009524. doi: 10.1371/journal.pcbi.1009524 (PMC8568149; doi:10.1371/journal.pcbi.1009524)
Supplement: Attachment — Submitted filename: Response to reviewers.pdf [file pcbi.1009524.s001.pdf]

Dear Dina,

Thank you for the opportunity to submit a revised version of our manuscript. We have addressed each of the reviewer comments as detailed below in point-by-point form, with changes to the manuscript marked in red. To summarise, we have clarified how our work unlocks an analysis pipeline for methylation data within Bioconductor, better explained how the spaghetti plot is generated and what is represents, implemented new functions for PCA and heatmaps in the latest version of *NanoMethViz* along with a vignette describing use of these new features and other general code improvements.

We hope that you will consider our revised work suitable for publication.

Yours Sincerely,
Shian Su

Reviewer #1

The authors have developed NanoMethViz, a tool for the visualization of modified nucleotides from nanopore data. I am quite familiar with this space, as I have developed a similar tool (methplotlib; doi.org/10.1093/bioinformatics/btaa093). The integration of NanoMethViz with the bioconductor ecosystem is highly valuable, especially in the context of further (statistical) analysis. Detecting nucleotide modifications from nanopore data is a fast moving field with many software tools and data formats, and there is clearly no one-size-fits-all approach. The use of NanoMethViz in an interactive R session is indeed valuable, compared to methplotlib which is purely command line based. The software appears well-documented and the visualizations of NanoMethViz are attractive and of high quality. As such NanoMethViz represents an important contribution to the field and wish to congratulate the authors with their work. The manuscript is well written and easy to follow.

Sincerely,

Wouter De Coster

I am not unbiased in this matter, but I have my reservations about how NanoMethViz is compared to existing tools in this space.

Response: We thank the reviewer for their positive feedback and careful consideration of our work.

(1) The 'Authors summary' states there is "a lack of software for effective visualization of methylation calls based on nanopore platforms", which is demonstrably incorrect. The introduction, however, is a more honest representation of the current state of the field, as the following is stated "There is currently no software in the R/Bioconductor collection for easily creating plots of methylation profiles".

Response: We have amended the text to refer more specifically to the lack of software for this kind of analysis in the R/Bioconductor ecosystem and have also highlighted the data wrangling steps that allow interoperability with other Bioconductor packages. We hope that this serves to highlight the long-read methylation analysis environment that our work unlocks within Bioconductor. The abstract now reads 'The lack of R/Bioconductor tools for the effective visualization of nanopore methylation profiles between samples from different experimental groups led us to develop the *NanoMethViz* R package.' We have also modified the Author summary to remove the sentence about the 'lack of software for effective visualization.'

(2) Some of the statements about methplotlib are also outdated, although these very well might have been correct at the time of writing the manuscript. Methplotlib similarly uses bgzip and tabix for efficient access to tabular files, and has PCA and pairwise correlation plots for a higher level assessment. Smoothening is done using a rolling window average. Interestingly enough, I also picked the GNAS locus as an example for my publication. There is absolutely no need for rivalry or competition between software developments and I consider NanoMethViz a valuable contribution to the field, but I would appreciate a more accurate representation (and citation) of tools with a similar scope.

Response: Thanks for pointing this out - we have reviewed the current *methplotlib* features and adjusted the text to more accurately reflect the current feature set. The new text on this reads as follows:

‘*Methplotlib* creates detailed plots of specific genomic regions, including a line plot of the methylation frequencies of individual samples, a heatmap of the methylation profiles on individual reads, and PCA as well as pairwise correlation plots for high level inspection of data.’

We 100% agree with you that GNAS is an interesting locus to visualize - we also highlighted it in previous work (see Figure 4b of Gigante *et al.* 2019, NAR, 47(8):e46).

(3) I am a bit surprised about the noisiness of the individual reads (as shown in the spaghetti plots). Can the authors comment on this, and perhaps provide a recommendation towards which coverage would be minimally required for accurate average methylation probabilities?

Response: The noise mostly exists in the non-control-regions, with significantly less noise in control regions. As such it may be that methylation state is less consistent outside of control regions.

New text has been added: “Smoothing is performed over the methylation probabilities reported by methylation callers. A smoothed value near 0.5 can therefore arise either because adjacent CpGs have opposite methylation status (confidently called as 0.99 and 0.01) or because the caller has low confidence in the interval (probabilities around 0.5). Therefore biological and technical noise are confounded in the spaghetti representation.” and “The spaghetti plots for individual reads allows visualization of methylation probabilities along single molecules; however, the data can appear noisy when plotted over large genomic regions, when coverage is high, and in regions with high site-to-site variation in methylation. In these placental samples, we see that there is a high level of variation in methylation probabilities outside of control regions and highly consistent signals within control regions.” to clarify the measurement being smoothed and interpretation of the information.

We defer to the conventional wisdom of 30x coverage recommended for WGBS analysis, and also the suggestion of Hansen *et al.* (2012) Genome Biology, 13:R83 that as low as 4x coverage could be sufficient for differential methylation analysis given advanced statistical methods. We have also performed non-rigourous subsampling analysis based on picking out a certain fraction of reads from the existing methylation table for the genes *Peg3* and *Meg3* to obtain varying coverage (ranging from 40x total (i.e. 20x per haplotype group) down to 2x total (i.e. 1x per group) as shown in the figure below.

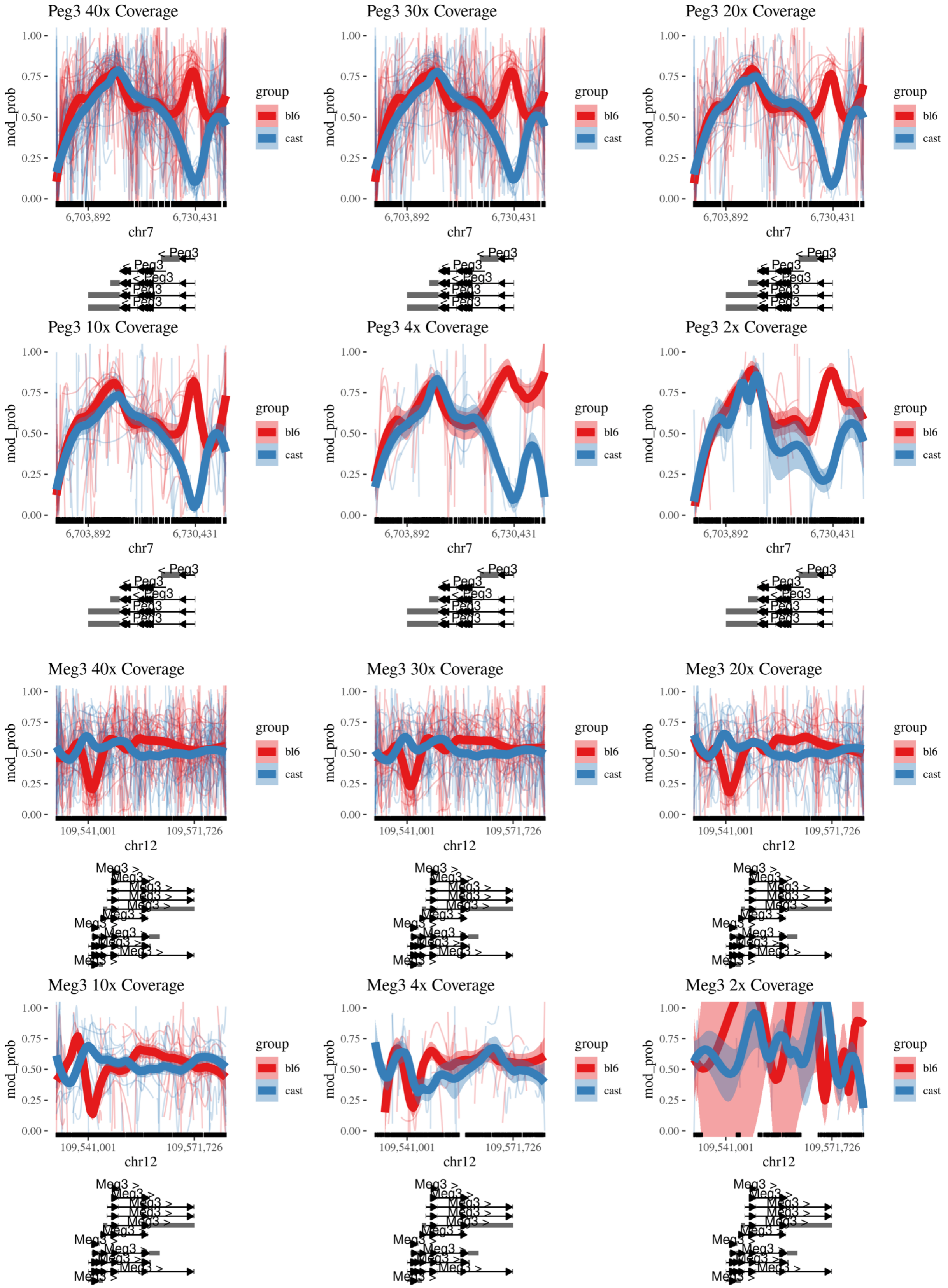
Peg3 40x Coverage
Peg3 30x Coverage
Peg3 20x Coverage
Peg3 10x Coverage
Peg3 4x Coverage
Peg3 2x Coverage
Meg3 40x Coverage
Meg3 30x Coverage
Meg3 20x Coverage
Meg3 10x Coverage
Meg3 4x Coverage
Meg3 2x Coverage
mod_prob
1.00
0.75
0.50
0.25
0.00
chr7
6,703,892
6,730,431
chr12
109,541,001
109,571,726
group
bl6
cast
Peg3
Meg3

It would appear that just over 10x total coverage (5x coverage per experimental group) is sufficient to reveal methylation patterns via the aggregated trend, and going above 20x total (10x per group) does not significantly change the information inferred from the trend lines, which is broadly in line with the recommendation in Hansen *et al.* (2012) above.

Reviewer #2:

In their manuscript “NanoMethViz: an R/Bioconductor package for visualizing long-read methylation data” Su et. al describe their newly developed Bioconductor package to visualize long-read methylation data. The package seems useful and is overall well described such that I would in principle recommend publication.

Response: We thank the reviewer for their constructive and positive feedback on this work.

Nevertheless, I have a few minor comments/suggestions that I think should be addressed:1. Shouldn’t the methylation signal in single reads as for example displayed in the Spaghetti-Plots be binary i.e. 0 (Not methylated) or 1 (Methylated). I realize that methylation likelihoods or probabilities are displayed, but in that case shouldn’t values near 0.5 be excluded as unreliable? So, does it really make sense to smooth these values? Along these lines, assuming a binary signal for single CpGs along a read (thin lines), is a line-plot really the appropriate way to display this. Wouldn’t one rather use a point-graph (each CpG per read gets a point). This would avoid the appearance of continuity where there is none (weird zig-zagging). The smoothed regression line aggregating “everything” is fine, though, since here continuity is indeed intended (similarly to what is done in conventional short read DNA methylation analysis).

Response: The true methylation state is indeed binary (either 0 or 1) and the spaghetti plot does not reflect this. However, it is a direct visualisation of the raw data, and is sufficiently informative when highlighting differentially methylated regions. We have tested a modified version of the spaghetti plots that binarises the methylation calls to 0 or 1 and applies smoothing to those values, for high coverage data it did not appear to make a significant difference in the final visualisation. The top two plots show the current method compared to if the calls were binarised, bottom two plots show the underlying data points overlaid, though the data points have snapped to the two extremes of the plot at 0 and 1, after smoothing it appears to not be significantly different to using the raw probabilities to smooth.

Peg3 Probabilities

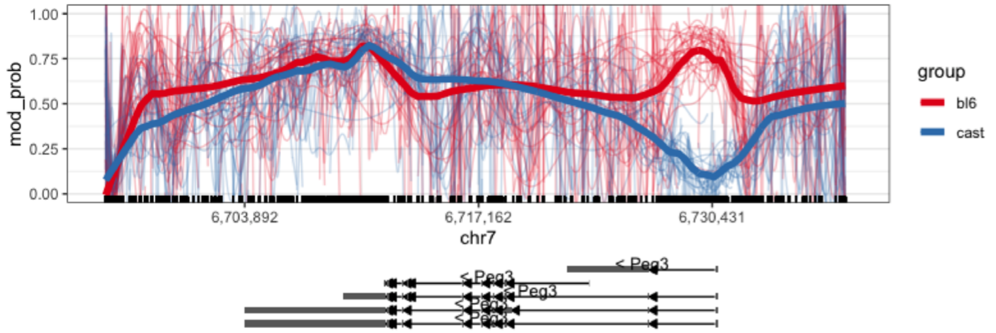

Peg3 Binarised

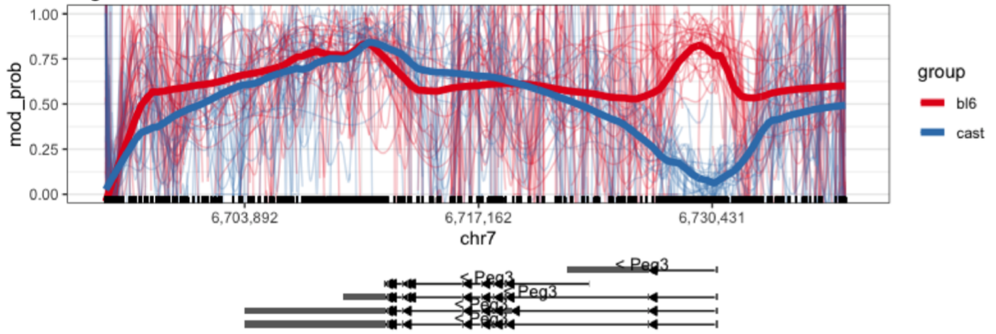

Peg3 Probabilities

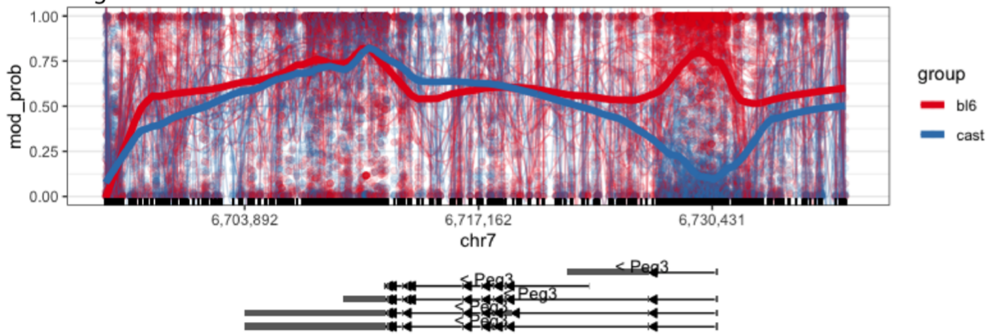

Peg3 Binarised

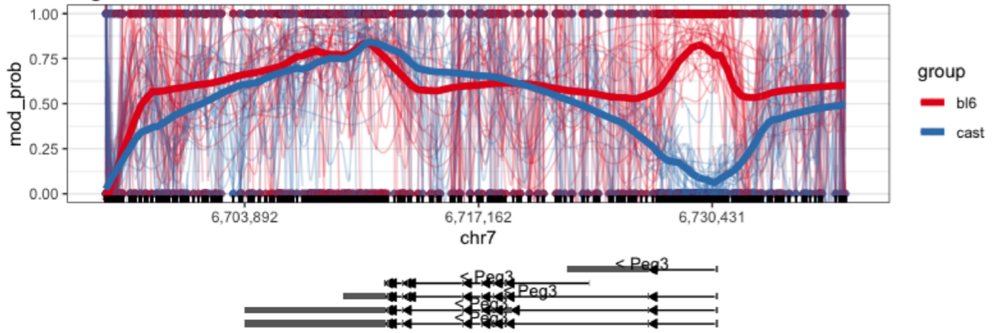

In some situations, such as in the plots below, where the region is roughly 4-5x coverage and without strong biological signal, binarising may cause all the data to snap to one extreme of the plot. In this case all information about the noise in the signal is lost.

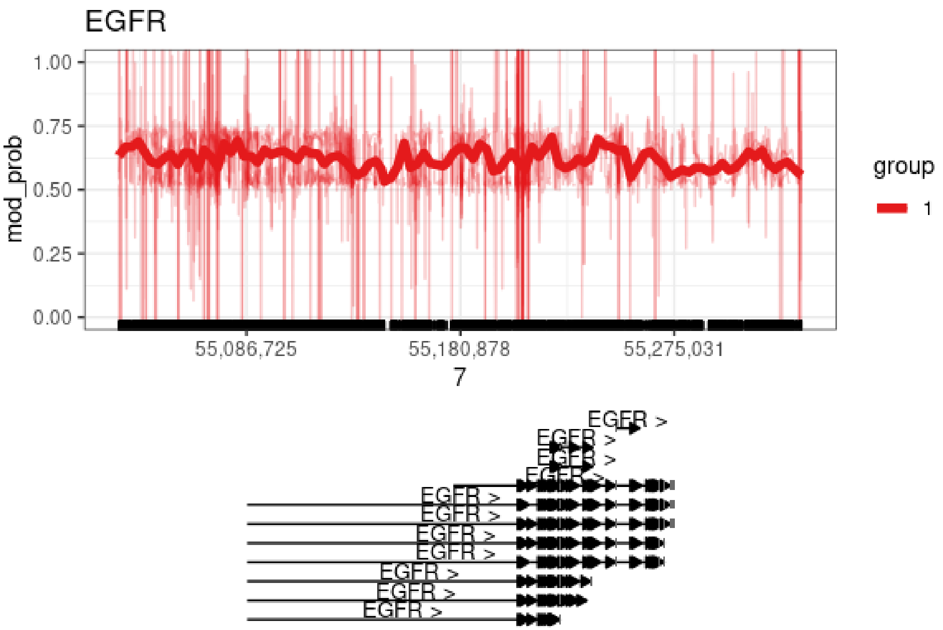

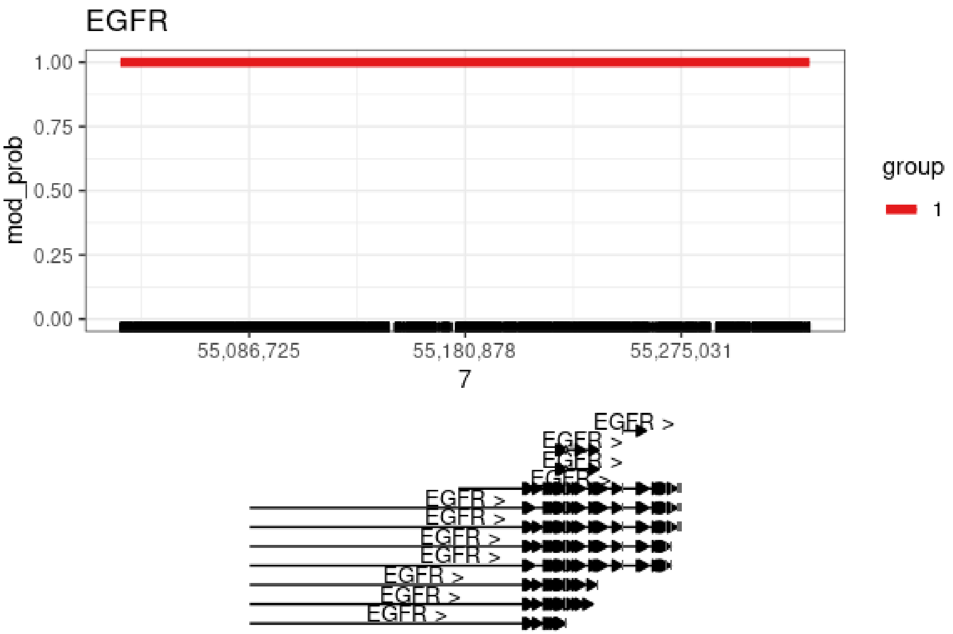

Under the current implementation, values near 0.5 indicate maximum uncertainty or adjacent cytosines with opposite methylation states, and could be filtered out from the display, but working out how to do this sensibly is a challenge. If a read contains some sites of high confidence, and others of low confidence, should the entire read be excluded or only the low confidence sites? Filtering the reads as a whole might lead to significant loss in data, while filtering only the low-confidence sites will create potentially problematic discontinuities in the plotting. We currently leave the question of filtering up to the users for now until we can come up with a more general approach.

We do however think that line graphs are reasonable given the autocorrelation present in methylation signal. A point graph is indeed a good alternative representation of the data, and to maintain the per-read linkage information in the data a heatmap for this purpose has been implemented in the latest developmental version of *NanoMethViz* (https://github.com/Shians/NanoMethViz, via the `plot_region_heatmap` and `plot_gene_heatmap` functions). It is our opinion that the smoothed curves are still easier to visually parse for large quantities of data, and that the heatmap will provide higher resolution insight after initial visual screening.

New text has been added: "Smoothing is performed over the methylation probabilities reported by methylation callers. A smoothed value near 0.5 can therefore arise either because adjacent CpGs have opposite methylation status (confidently called as 0.99 and 0.01) or because the caller has low confidence in the interval (probabilities around 0.5). Therefore biological and technical noise are confounded in the spaghetti representation." and "The spaghetti plots for individual reads allows visualization of methylation probabilities along single molecules; however, the data can appear noisy when plotted over large genomic regions, when coverage is high, and in regions with high site-to-site variation in methylation. In these placental samples, we see that there is a high level of variation in methylation probabilities outside of control regions and highly consistent signals within control regions." to clarify the measurement being smoothed and interpretation of the information.

2. It is not clear how MDS can be performed without loading all of the data at once, a necessity to allow non HPC analysis as discussed by the authors. A bit more background on how this is achieved would be great.

Response: The MDS plot is generated by first performing blockwise transformation of the full methylation data (tens of GB worth of data in this instance) into a count matrix (<1GB) that is used for plotting. This allows the MDS plot to be created under memory constraints, which is addressed (albeit indirectly) in the current text with: 'Conversion is performed using block-wise streaming algorithms...'

3. Would it be possible to also enable PCA or even biplot visualizations? This would have the advantage of instantly getting an estimate of the main axis of variability as well as the driving molecular features (methylation patterns).

Response: Yes, these would both be great additions to *NanoMethViz*. The PCA plot has been added to the latest developmental version of *NanoMethViz* on Github via the `plot_pca` function, and the biplot will be explored as an extension.

4. One more analysis/visualization to have (as a bonus) would be to calculate and visualize methylation correlations along the length of a read (i.e. correlation between two CpGs in dependence of their distance).

Response: We agree that it would be interesting to add this type of analysis/visualization feature to *NanoMethViz*. Unfortunately, our present data structure is not set up to allow data

queries on reads, only on genomic intervals. A limited form of this analysis could be done by querying a region, and separating the data by read names, but we would not be able to guarantee that data on a read is not lost by falling outside the query region. However, this would be sufficient for use cases that are interested in behaviour in a specific region of interest rather than specific reads.
